# Human rights and mental health in post-apartheid South Africa: lessons from health care professionals working with suicidal inmates in the prison system

**DOI:** 10.1186/s12914-017-0136-0

**Published:** 2017-10-12

**Authors:** Jason Bantjes, Leslie Swartz, Pieter Niewoudt

**Affiliations:** Department of Psychology, Stellenbosch University, Private Bag X1, Matieland, 7602 South Africa

**Keywords:** Human rights, Mental health, Post-apartheid, South Africa, Suicide prevention, Prisons

## Abstract

**Background:**

During the era of apartheid in South Africa, a number of mental health professionals were vocal about the need for socio-economic and political reform. They described the deleterious psychological and social impact of the oppressive and discriminatory Nationalist state policies. However, they remained optimistic that democracy would usher in positive changes. In this article, we consider how mental health professionals working in post-apartheid South Africa experience their work.

**Methods:**

Our aim was to describe the experience of mental health professionals working in prisons who provide care to suicidal prisoners. Data were collected from in-depth semi-structured interviews and were analyzed using thematic content analysis.

**Results:**

Findings draw attention to the challenges mental health professionals in post-apartheid South Africa face when attempting to provide psychological care in settings where resources are scarce and where the environment is anti-therapeutic. Findings highlight the significant gap between current policies, which protect prisoners’ human rights, and every-day practices within prisons.

**Conclusions:**

The findings imply that there is still an urgent need for activism in South Africa, particularly in the context of providing mental health care services in settings which are anti-therapeutic and inadequately resourced, such as prisons.

## Background

At the height of the political turmoil and state repression that was a hallmark of the last decade of apartheid in South Africa, a number of mental health professionals commented on the impossibility of conducting truly ethical mental health practice in an oppressive social context [1–3]. Commonly, they envisaged a post-apartheid future in which there would be a more equitable distribution of wealth, and a more just society, conducive to human flourishing and psychosocial well-being [4]. It has been more than 20 years since the advent of democracy in South Africa and, while some advances have been made towards greater equality and justice, there are still considerable structural, economic and socio-political factors which hamper the delivery of mental health care and provide less than optimal conditions for psychological well-being [5, 6]. The question arises, then, of how mental health professionals working in contemporary South Africa experience their work, and how they think about the social, economic and political constraints they face. In this article, we present and discuss data gleaned as part of a larger study on the challenges of suicide prevention in South Africa. In this aspect of the work, we were interested in how mental health professionals working in the correctional system (prisons) experience their work in relation to dealing with suicidal prisoners. The prison system is an especially useful context within which to explore these issues; in South Africa, as elsewhere in the world, rates of suicidal behavior are high among prison populations, with suicide being the most common cause of death among inmates [7–9]. In addition, the prison system in South Africa is woefully under-resourced, with overcrowding, gangsterism and violence within prisons being as bad as, if not worse, than they were under apartheid [10–12]. Our data draw attention to the complex ethical and human rights challenges mental health professionals in post-apartheid South Africa face when attempting to provide psychological care in settings where resources are scarce and where the environment is anti-therapeutic. We consider the human rights and advocacy implications of our findings, within the context of existing legislative frameworks in South Africa.

### Post-apartheid South Africa

South Africa became a democracy in 1994, with Nelson Mandela, a global icon, as president, and with a vision to create a more just society. The South African Truth and Reconciliation Commission, a public performance of national reckoning and healing, was hailed internationally as something of a model for post-conflict societies [13]. South Africa was celebrated, not just for overcoming its racist past, but also for having the courage to face the pain and suffering that apartheid caused, and for engaging in the difficult work of reconciliation [14, 15]. Some remarkable stories of reconciliation and psychological healing have emerged from South Africa, including tales of direct collaboration for a just society between family members of those who were killed as part of the political struggle, and the killers themselves [16–18]. Nobel Laureate, Archbishop Emeritus Desmond Tutu has espoused a theology of forgiveness, arguing that forgiveness of enemies and oppressors takes profound psychological work, but also provides a key route for psychological and spiritual healing [19]. The language of liberation, justice, forgiveness, reconciliation and collaboration has become part of the political zeitgeist of the “new” South Africa.

Alongside these achievements, there has, however, been a more dystopian view of South African society. Democracy has certainly benefited many, including a relatively small Black middle-class elite, but most South Africans continue to live in poverty and in circumstances which are far from optimal in terms of health and human development [20]. As in many other parts of the world, the gap between rich and poor has widened since 1994, and unemployment rates remain very high [21]. There is wide-scale corruption [22], and the prevalence of interpersonal violence, including gender-based violence and murder, are amongst the highest, if not the highest, globally [23, 24].

A contemporary re-reading of the anti-apartheid psychology writings about the impact of apartheid on human development and well-being reveals that many of the issues psychologists were concerned with remain, and in some cases, have been exacerbated since 1994 [25]. Poverty, inequality, violence, and poor education, for example, remain key features of contemporary South Africa [21, 23, 24]. Issues of substance abuse are probably worse than before, with South Africa emerging as more of a global player in the illegal drug trade [25]. During the apartheid era, some mental health professionals looked forward to an improved future under democracy; although there have been important social changes, many contemporary mental health professionals in South Africa continue to work in settings of endemic social problems without the hope or expectation that a radical social transformation is likely to occur. The South African prison system is an apt example of one such setting in which mental health professionals work under conditions which are far from optimal, are distinctly anti-therapeutic, and in which there is little expectation of reform, despite a legislative framework which espouses the importance of protecting human rights [26].

### The South African prison system

Prisons in South Africa are under the control of the Department of Correctional Services (DCS) which obtains its legal mandate from the Correctional Services Act (Act 111 of 1998), Criminal Procedure Act (Act 51 of 1977), Child Justice Act (Act 75 of 2008), Promotion of Administrative Justice Act (Act 3 of 2000), and Prevention and Combatting of Torture of Persons Act (Act 13 of 2013). The DCS is also governed by the 2005 White Paper on Corrections, the 2014 White Paper on Remand Detention Management in South Africa [27], and the Constitution of the Republic of South Africa (Act 108 of 1996) [28]. This legal framework explicitly requires the DCS to provide “a safe, secure and humane environment” [29] (para. 2), and to safeguard the rights of all offenders, including the right to human dignity, freedom and security of the person, satisfactory accommodation, and adequate medical care. The DCS has fallen woefully short of its statutory responsibilities to protect the rights of prisoners and create safe and humane prisons [26].

There is a growing body of literature describing the inhumane and unsafe conditions within South African prisons. Overcrowding and resource constraints are major concerns, and are associated with elevated levels of psychological stress, psychiatric illness, interpersonal violence, physical assault and sexual abuse within South African prisons [30–33]. Benatar [11] has drawn attention to how overcrowding leads to a lack of rehabilitation programs, and extremely limited and often non-existent recreational or work opportunities in South African prisons. Inmates report that they have easy access to a range of substances within local prisons [34, 35]. Rates of interpersonal violence and sexual assault are also alarmingly high within South African prisons, mostly as a result of the nefarious influence of gangs [12, 30–32]. Furthermore, rates of psychopathology are high among prisoners; Naidoo and Mkize [35] report that 55.4% of offenders met DSM-IV diagnostic criteria for an Axis 1 disorder, and Prinsloo [33] found that 78.4% of offenders with sentences of more than 25 years had diagnosable mental conditions. There is a significant mental health treatment gap, with very few prisoners receiving the psychological and psychiatric care they require [35].

### Suicide in South African prisons

Approximately 40% of all deaths in South African prisons are due to suicide [36], making suicide the primary cause of unnatural death among prisoners [8]. There were 61 suicides in correctional centers in South Africa during 2015 [37], which yields a prevalence rate of approximately 36:100,000, almost three times the national suicide prevalence rate [38]. Suicide prevention in prisons is a human rights issue as well as a public health concern [39], yet the problem has received very little attention.

International literature suggests that there are a number of contextual and systemic factors within prisons that contribute to suicide. These factors include: high rates of psychopathology; poor provision of mental health care; the lack of purposeful and stimulating activities (such as work and education); segregation and isolation; victimization; and violence [9, 40]. It is within this context that we were interested in exploring the challenges health professionals working in South African prisons face when attempting to provide care to suicidal inmates.

## Methods

We adopted an exploratory qualitative research methodology to investigate the experiences of health professionals working in South African prisons and their perceptions of the challenges to suicide prevention in this setting. We purposefully sampled a group of 10 health professionals (doctors, psychiatrists, psychologists, nurses and social workers) from one medium- and one maximum-security correctional center in Cape Town, South Africa. The maximum-security correctional center is home to approximately 7000^1^ male and female awaiting trial and sentenced offenders, housed within facilities built to accommodate approximately 4300 and staffed by approximately 1300 personnel. According to the Department of Correctional Services [37], the medium-security correctional center has a population of approximately 990 sentenced and 1165 unsentenced male offenders, and a staff of approximately 511. There is 24-h a day nursing services available, a private psychiatrist visits the center once a month for consultation with inmates in need of this services, a medical officer visits the center four times a week, and emergency dental services are rendered once a week. The center also has a number of social workers, each of whom has a case load of approximately 240 inmates.

All health care professionals (*n* = 19) working in these two correctional centers were invited via email to participate in the study, of whom 10 agreed to participate (yielding a 53% participation rate). The age distribution of the sample was 27–51 years, with a mean age of 39 years (SD = 12). The sample consisted of five psychologists, three medical nurses, one social worker and one psychiatrist. Their work experience within correction centers ranged from six months to 13 years, with an average of 6.2 years’ experience. The ethnic composition of the sample was diverse; five of the participants self-identified as White, four identified as Colored,^2^ and one identified as Black.

Data were collected via in-depth semi-structured interviews conducted face-to-face. During these interviews, participants were asked to describe their experience of providing care to suicidal prisoners. Examples of questions included in the interview were: *Tell me about your experience of working with suicidal prisoners? What can you tell me about the conditions under which you work? What is your perception of the factors that hinder or promote suicide prevention within your setting? What suggestions do you have for suicide prevention in local correctional centers?* The interviews, which lasted approximately 50 min each, were audio recorded and transcribed. Thematic content analysis was employed to code the data using an inductive approach and the process outlined by Braun and Clarke [41]. The data were analyzed independently by two authors, and then codes were compared. In an effort to improve the trustworthiness of the findings, the interviewer repeatedly checked with participants to clarify what participants meant during the interviews and to confirm that what participants reported was being correctly interpreted.

Two superordinate themes emerged from the analysis: (1) systemic and contextual factors that impede suicide prevention; and (2) participants’ experience of the impossibility of providing adequate care to suicidal inmates in a resource scarce anti-therapeutic environment. The findings of the first theme and the implications for suicide prevention in prisons have been reported elsewhere. In this article, we report on the second of these themes and focus our discussion on the human rights implications of our findings for advocacy. Permission to conduct the study was obtained from the relevant university research ethics committee (Health Sciences Research Ethics Committee Reference N12/05/029) and the Department of Correctional Services. Written informed consent was obtained from participants prior to data collection. All data were securely stored and the identity of the participants protected by employing pseudonyms.

## Results

Participants said they were regularly called on to provide care to suicidal prisoners. Joey said he encountered suicidal inmates *“daily”* and Des said, *“I would say frequently, so every time I have a session at the prisons, there is at least one person that exhibits such behavior or ideation.”* They said that some of the prisoners in their care had a long history of mental health problems and suicidal behavior, dating back to before incarceration, while others became suicidal as a result of the stress and trauma of incarceration. Participants also reported that prisoners under their care had completed suicide. For example, Abi said, *“…we recently had a death where someone hanged himself.”* Participants identified a number of factors that hindered their capacity to provide care to suicidal prisoners and they described how they felt compromised as mental health professionals working in an anti-therapeutic environment, with a lack of support, collaboration and resources.

### Prison is not a therapeutic environment

Participants explained that prisons were primarily organized to focus on security, control and containment; as such they are not therapeutic environments. They described their working environment as *“difficult”* and *“anti-therapeutic”* and said that the prison context was *“hopeless”* and *“alien”*. Joey, for example said, *“Prison is something else – a different world!”, and Lee said, “It’s a very hopeless environment.”*

Participants said it was often difficult, if not impossible, to institute psychological interventions and provide optimal levels of care within the prison environment. Lee, for example, said, *“What happens is people are so overworked and stressed, that intervention creates difficulties you know…”* Lee went on to explain that these *“difficulties”* were exacerbated by problems such as *“overcrowding and understaffing”* in prisons.

Participants noted that health facilities within prisons are equipped to treat physical and medical conditions, rather than provide integrated psycho-social or psychiatric care. Lee described this by saying:

*“The correctional services are a little more geared to treating people with more normal physical, not mental, health symptoms, and once they then become severe they would, say, transfer the person to hospital…”*

Lee recounted an example of this by saying:

*“Like, for example, this one person that I work with who, his arms are like completely cut, like everywhere on his arms, he has tried to cut out his heart, his heart is like all scarred around and like – he is psychotic and he just harms himself. So, somebody like that I feel absolutely clear we can’t provide the necessary therapeutic level.”*

### Lack of collaboration, integration and support

Participants spoke of the importance of teamwork and collaboration between staff members as well as the value of standardized procedures and protocols in order to keep at risk prisoners safe. They said it was also difficult to maintain a standard of care when attending to suicidal inmates because they were dependent on other staff, such as wardens, to play their part in monitoring prisoners and ensuring their safety. Des described how the standard of care was highly variable and unpredictable, by saying:

*“…obviously, that (standard of care) is affected by who is on duty. Obviously, if that person (who is competent and reliable) is not on duty, I cannot bring them on board. And even if I bring them on board and they go off duty another day, then that task will not be taken care of and will fall to the general system – so it is erratic at best.”*

Participants said that they often felt isolated and unsupported as it was not always possible to work as part of a multidisciplinary team or provide integrated care. They said they were often required to perform their duties in isolation, without supervision and with no support. Sue explained:

*“There is no teamwork; we are very isolated in terms of being supported by other health professionals.… We have no input from that (other team members). The unit managers have no clue what these guys (offenders) are doing half the time, they don’t even know who they are.”*

Similarly, Lee said:

*“Which is also why it is difficult to work as a multidisciplinary team, you feel like you going into someone else’s territory and people are very territorial.”*

Participants said they encountered obstacles when attempting to transfer prisoners to state psychiatric care because of bed shortages in tertiary psychiatric hospitals. In part, the challenges participants face when trying to transfer mentally ill prisoners into specialist psychiatric facilities, is the consequence of legislation which makes the medical care of prisoners the responsibility of the Department of Correctional Services, rather than the Department of Health. Psychological and psychiatric services in South African prisons exist distinct from the health care system, with a lack of meaningful integration and functional collaboration between these two parallel systems. Joey described this lack of collaboration by saying, *“There is no sense of team work.”* Lee echoed this, saying:

*“It’s very dependent on the members (wardens) responding appropriately, and also the hospitals are overcrowded, or there is a shortage of professionals in nursing staff, so suicide watch is quite a problem there. I think it (the failure to provide adequate care) just happens on the most basic level.”*

Participants spoke of their need for support and guidance when dealing with suicidal offenders. In this context, some participants said a standardized procedure and assessment process would be helpful. Xolisa, for example, said:

*“I would like a form that can tell me exactly, that if they answer this to that question, they will definitely do it and it’s not about them being manipulative. Because sometimes that is how we see it as.”*

### Lack of resources

Participants said that they had difficulty providing optimal care and fulfilling their professional obligations because of a lack of basic resources, such as psychiatric medication. Lee said:

*“It is, for example, very difficult to get access to the drugs (medication) – so I think you do see a lot of untreated depression…ultimately we rarely have the resources to do a complete, like suicide watch… Our resources are a lot more limited.”*

Participants said the shortage of mental health care staff in prisons put them under pressure and created time constraints. They acknowledged that this sometimes hampered their ability to conduct thorough suicide risk assessments and provide appropriate monitoring and follow up care. Lee said:

*“I really don’t have time to do a long history so it has to be quite obvious stuff, it can’t be more than that because I only get to see people for 40 minutes.”*

Sue echoed this experience saying:

*“Maximum I can see them is once a day, and I have nobody I can just sort of phone and say, listen how is so and so doing? There is no access to it (support and monitoring) and obviously in a private environment it’s different; you can call on the family, you can put all different sort of procedures in place to keep them safe, but we can’t do that here.”*

Participants said that they felt ethically compromised working in prisons and believed they would be better able to prevent suicides with access to more resources. Lee said:

*“I think that in a more highly resourced environment more can be done to prevent it (suicide).”*

Sue echoed this saying:

*“So as far as ethical duties are concerned, I think the number one biggest problem that we are sitting with, why nothing gets complied with and no follow through, there is no follow through, there is no money, there is no money pumped into the prisons. Society has locked up these prisoners and thrown away the key; they don’t want to know, and as a result, the wardens don’t want to know, and they don’t get paid enough to want to know, and if they do get paid enough there is not enough of them. There is a moratorium on hiring wardens, at the moment…and there is a serious shortage, so we don’t have the means to keep these guys (prisoners) safe.”*

### Anxiety, impotence, sadness and hopelessness

Participants articulated how working within the prison system and within such a resource constrained context, precipitated high levels of stress and anxiety. They said they felt compromised in this setting and could not provide optimal levels of care. They said this made their work difficult and frustrating. Sue said:

*“The numbers are just too massive to vaguely give the guys individual attention, so it is literally left up to us, and the questions that we can ask in the room at that time, and in total isolation from anything else, and that is what probably makes it the biggest problem that we face apart from anything else… And obviously, a huge amount of anxiety where that is concerned, we all do.”*

Sue went on to elaborate on her feelings of anxiety and explain how it contributed to feelings of sadness, by saying:

*“…it does not make me angry, it does not irritate me. It does make me a little anxious, and if anything, it does make me very sad.”*

Participants described how the cumulative impact of this anxiety contributed over time to feeling hyper-vigilant and constantly on guard. They said this made it difficult for them to relax and consequently they carried their work with them even when they went home. Sue described this experience saying:

*“The faces like that you don’t just leave, they kind of stick with you in your mind. I would like to say I leave them at prison when I go home, but I don’t.”*

Participants also described how powerless and overwhelmed they felt by their work. They said that providing care to suicidal inmates was challenging, emotionally provocative, and that it often left them feeling helpless because they did not believe they could do anything to address the structural factors contributing to the problems their patients’ experienced. Sue articulated these feelings of impotence, saying:

*“…as I say, that helplessness and powerlessness, we feel a lot of that as well.”*

In large part the feelings of anxiety, hopelessness and powerlessness reported by participants seemed to be a function of their experience of working in a resource constrained environment, without adequate support, and with poor communication. They said that they would feel more able to meet the challenge of suicide prevention if there were more mental health professionals working within prisons, if they had access to more support and training in suicide prevention, and if systems were established to promote communication and team-work. For example, Sue said:

*“We need resources. We need more psychologists in the prisons. But we also need more education. We need more members (wardens). We need all of those things, but we also need a team. We need the health professionals to communicate with each other. We need the unit managers to communicate with us. We need unit managers allocated to solely help alone, that work with the offenders, that are educated, that know what it means when somebody is suicidal. They don't have a clue, so the education is not there. The resources are not there. The manpower is not there, and there is no teamwork. There is a lot more teamwork needed.”*

## Discussion

The data we collected for this study make for depressing reading – mental health professionals in the prison system report feeling overwhelmed, unsupported, compromised and impotent to make the changes they believe important for optimal mental health and suicide prevention. It is clear from our data that these are professionals working under very difficult circumstances. These findings are not new. Previous research focused on the experiences of health care professionals working in prisons, has highlighted how frustrating and challenging it is to provide care and establish a therapeutic relationship within an environment where there is a distinct tension between a punitive culture of custody and a culture of care and rehabilitation [42, 43]. This is not surprising given that prison is designed with punishment, correction and rehabilitation to the community in mind and these goals may conflict with the aims of providing psychiatric care and preventing suicides [44]. Our participants’ call for greater teamwork and more support to perform their functions, is echoed in international literature which asserts that effective treatment for mentally ill prisoners often entails services provided by a multidisciplinary treatment team that includes correctional officers [45]. Multidisciplinary teams can effectively address complex mental health and social care needs in prisons [46] and would seem to be an important component of suicide prevention.

Our participants also drew attention to the need to address contextual factors within the prison system and to create a caring culture in which prisoners feel safe. The importance of creating communities of care within prisons and establishing systems for screening and appropriate referrals of mentally ill prisoners have been highlighted in studies from high-income countries [47]. There is growing acknowledgment in the international literature of the need for correctional centers to be transformed into “psychologically informed planned environments” [48] (p. 84) and “therapeutic communities that target specific behaviors, such as drug and alcohol abuse and violent behavior” [49] (p.4), in an attempt to bridge gaps between therapy and custody. This move towards creating humane correctional centers which seek to rehabilitate rather than punish offenders is entirely consistent with the legislative framework and human rights discourse in contemporary South Africa. However, our findings draw stark attention to the considerable gap between current policy and actual practice within South African prisons. Crucially our findings also highlight the despondency and lack of hope for meaningful change felt by the health care workers’ who participated in our study. The tone of despair, frustration, and powerlessness expressed by participants in this study is very strong. One of the paradoxes of the practice of psychology and similar disciplines under apartheid was that there was always an imagined better, democratic future to look towards and to aspire to. Currently, mental health professionals are working within a democratic dispensation, but without the reasonable hope of major positive social change on the scale of the transition from apartheid to democracy. Participants are operating in the context of a very stressed health care system and also in the context of a society in which much of the hope for substantial social change in a range of areas, including health care, has dissipated as new challenges to a young democracy have emerged. In this regard, Harris et al. [50] (p. 584) note in their study of attempts to reform aspects of primary health care in South Africa:

*The notion of restorative practices assumes that those in authority have scope to shape the social world, to manufacture the conduct of those in their power, simply by virtue of how they practice their authority… Democratically transforming outdated modes… [of care] requires a change in the conduct of providers as much as of patients, and with this, a better understanding of why and how providers themselves are done to, for, or with.*

As these authors note, the question of providing appropriate care in a democratic dispensation depends not only on the behavior of clinicians but also on how the clinicians themselves are positioned and treated within a system of care. Our participants are clearly seriously constrained by the lack of an enabling context and adequate resources, despite the progressive policy formulations which exist on paper, as we have mentioned in the introduction.

Clearly, the situation of these practitioners underscores the need for clinicians to look “beyond the 50-min hour” (as Goodman et al. [51] put it) to improve mental health and help prevent suicidal behavior in the prison population. This will require advocacy and sustained effort to transform practices and procedures within South African prisons. This kind of activism and engagement requires time, energy, and the opportunity to reformulate and broaden thinking about the scope of practice of mental health work in South Africa, not only in prisons but also more generally within the country’s  health care system. Furthermore, it is clear from the data that this kind of time, energy, and space to organize and advocate is not realistically available to these practitioners.

There are two fundamental implications of our findings. First, it is important to recognize and acknowledge the difficulties and challenges faced by mental health professionals working in the post-apartheid context. Countries like South Africa have been termed “post-conflict” societies, but in truth South Africa is far from being a post-conflict context. It is true that the war for liberation from the oppressive nationalist policies of the former government have been fought and won, but apartheid has not been replaced by conditions of peace and equality. Post-apartheid South Africa is a context in which there continue to be endemic social problems that create conditions of oppression and violence. Second, given the realities faced by our participants, it is incumbent on South African mental health professionals to begin to formulate new ways of engagement in activism in the service of improved mental health. This activism and advocacy is arguably part of good mental health practice in all contexts [52, 53], but is possibly especially important in contexts, like South Africa, where resources are scarce [54]. Crucially this advocacy role should not just fall to mental health care professionals who are currently employed within the overburdened and under-resourced public service system; it is a responsibility that needs to be taken up by all mental health professionals in the country. In this regard, there is already important work emerging from South Africa in the field of global mental health, with a number of new and exciting low-cost interventions being developed for use in poorly-resourced contexts [55, 56]. This work often acknowledges the broader social context of inequality and exclusion, but it is also clear that for mental health in prisons and in other poorly-resourced sectors of South Africa to improve, better social conditions and greater equality in society will be necessary.

## Conclusion

It is time for a new activism around mental health in South Africa which works to practically engage with many of the same social challenges raised by anti-apartheid psychologists. The struggle for justice does not end with a change of regime and a rewriting of policy and legislation. Though this issue has been acknowledged in terms of the struggle for health in general in South Africa [20], much more needs to be done in terms of mental health and human rights on a broad scale in the post-apartheid context. This is important not only for suicide prevention in prisons, but also for creating conditions which are more broadly conducive to psychological well-being and mental health in South Africa. Mental health professionals have a vital role to play in advocating for these changes and helping to reform every-day practices which promote human rights and psychological well-being.

## Footnotes

1 This number fluctuates daily since the prison serves as a remand center for individuals awaiting trial.

2 “Coloured” is an official term, originally defined by the South African government in 1950, which continues to be used to denote people of mixed European (“White”) and African (“Black”) or Asian ancestry.
